# Graded Heterojunction Improves Wide-Bandgap Perovskite for Highly Efficient 4-Terminal Perovskite/Silicon Tandem Solar Cells

**DOI:** 10.34133/research.0196

**Published:** 2023-07-17

**Authors:** Wenming Chai, Lindong Li, Weidong Zhu, Dazheng Chen, Long Zhou, He Xi, Jincheng Zhang, Chunfu Zhang, Yue Hao

**Affiliations:** ^1^ State Key Discipline Laboratory of Wide Band Gap Semiconductor Technology & Shaanxi Joint Key Laboratory of Graphene, School of Microelectronics, Xidian University, Xi’an, Shaanxi 710071, China.; ^2^ Xi'an Baoxin Solar Technology Co., Ltd., Xi'an, 710071, China.

## Abstract

Wide-bandgap (WBG) perovskite solar cells (PSCs) are essential for highly efficient and stable silicon/perovskite tandem solar cells. In this study, we adopted a synthetic strategy with lead thiocyanate (Pb(SCN)_2_) additive and methylammonium chloride (MACl) posttreatment to enhance the crystallinity and improve the interface of WBG perovskite films with a bandgap of 1.68 eV. The excessive PbI_2_ was formed at grain boundaries and converted into MAPbI_3−*x*_Cl*_x_* perovskites, which are utilized to form the graded heterojunction (GHJ) and compressive strain. This is beneficial for passivating nonradiative recombination defects, suppressing halide phase segregation, and facilitating carrier extraction. Subsequently, the device with GHJ delivered a champion efficiency of 20.30% and superior stability in ambient air and under 85 °C. Finally, we achieved a recorded efficiency of 30.91% for 4-terminal WBG perovskite/TOPCon tandem silicon solar cells. Our findings demonstrate a promising approach for fabricating efficient and stable WBG PSCs through the formation of GHJ.

## Introduction

Perovskite solar cells (PSCs) have attracted substantial attention due to their outstanding properties and potential as the next-generation photovoltaic technology [1–6]. The single-junction PSCs have already achieved a certified power conversion efficiency (PCE) of 26%, which is close to the efficiency of single-crystalline silicon solar cells [7,8]. To surpass the Shockley–Queisser limit, researchers have proposed using tandem devices by connecting multiple single cells in series [9–12]. One of the most promising approaches for tandem solar cells is to use a wide-bandgap (WBG) perovskite (approximately 1.7 eV) as the top cell and a silicon bottom cell [12–15]. Therefore, high-quality WBG film is crucial for achieving efficient tandem devices.

WBG perovskites are typically produced by replacing iodide with bromide or substituting monovalent cations with cesium [16,17]. However, under illumination, this can lead to phase segregation and large open-circuit voltage (*V*_OC_) deficits, which can negatively impact device performance [14,18–21]. Researchers have developed several strategies to improve WBG perovskite films and enhance device efficiency. One of these approaches involves using organic molecules to modify the interface and improve energy level alignment [22,23]. Huang et al. [24] employed indene-C60 bisadduct-trans3 (ICBA) to reduce energy disorder in WBG PSCs and obtained a recorded PCE of 18.5%. Liu et al. [25] designed a self-assembled monolayer to minimize energy losses at the interface and achieved a high *V*_OC_ of 1.25 V in WBG PSCs. However, organic molecules have the disadvantages of inferior stability and high cost. Additionally, the long-chain or aromatic molecules were used to form low-dimensional perovskite and suppress ion migration [16,26–28]. For example, Liu et al. employed surface reconstruction and bifacial passivation method to minimize nonradiative recombination in 1.65-eV bandgap PSCs by posttreatment with methylammonium thiocyanate and phenethylammonium iodide [14]. A thin Ruddlesden–Popper perovskite layer was formed to create a hybrid 2-dimensional (2D)/3-dimensional heterostructure in WBG PSCs (1.72 eV) with the *n*-butylammonium bromide (BABr), which exhibited a remarkable PCE of 19.4% and *V*_OC_ of 1.31 V [29]. Yu et al. [30] introduced a 2D additive of 4-fluorophenylethylammonium iodide to simultaneously improve perovskite crystallization and passivate defects. Although these 2D additives can improve *V*_OC_ and stability, the carrier transport is limited because of their electrically insulating nature [31,32].

It is common that lead thiocyanate (Pb(SCN)_2_) was applied to improve crystallization and increase grain size to 1 μm in WBG perovskite films [33]. Unfortunately, the Pb(SCN)_2_ can induce excess PbI_2_ formation and damage device performance. Many researchers employed the solvent annealing process to avoid excess PbI_2_ formation and form a perovskite homojunction [4,34,35]. Furthermore, the incorporation of phenethylammonium iodide and Pb(SCN)_2_ could effectively enhance the crystallinity of WBG perovskite films and inhibit the excess PbI_2_ [36,37]. However, the 2D perovskite simultaneously was formed on the surface and affected charge transport. Therefore, it is challenging to simultaneously suppress the ion migration and improve carrier transport.

In this study, we have developed a surface in-situ restructure strategy to construct the graded heterojunction (GHJ) by introducing a conventional additive of Pb(SCN)_2_ and methylammonium chloride (MACl) posttreatment. The Pb(SCN)_2_ additive was utilized to enhance crystallization and form excessive PbI_2_ at grain boundaries. Subsequently, MACl posttreatment was employed to react with excessive PbI_2_ and transformed into MAPbI_3–*x*_Cl*_x_* perovskite, which can form GHJ and compressive stress on the surface. These benefits improve energy level alignment and suppression of ions migration, resulting in low nonradiative recombination and enhanced carrier transport. Consequently, the optimal WBG PSC achieved an impressive PCE of 20.30% and outstanding stability in ambient air and at an elevated temperature. Furthermore, we achieved a recorded efficiency of 30.91% in 4-terminal (4-T) perovskite/silicon tandem devices. The study demonstrates the effect of GHJs on promoting the performance and stability of WBG PSCs.

## Results

**Fig. 1.**
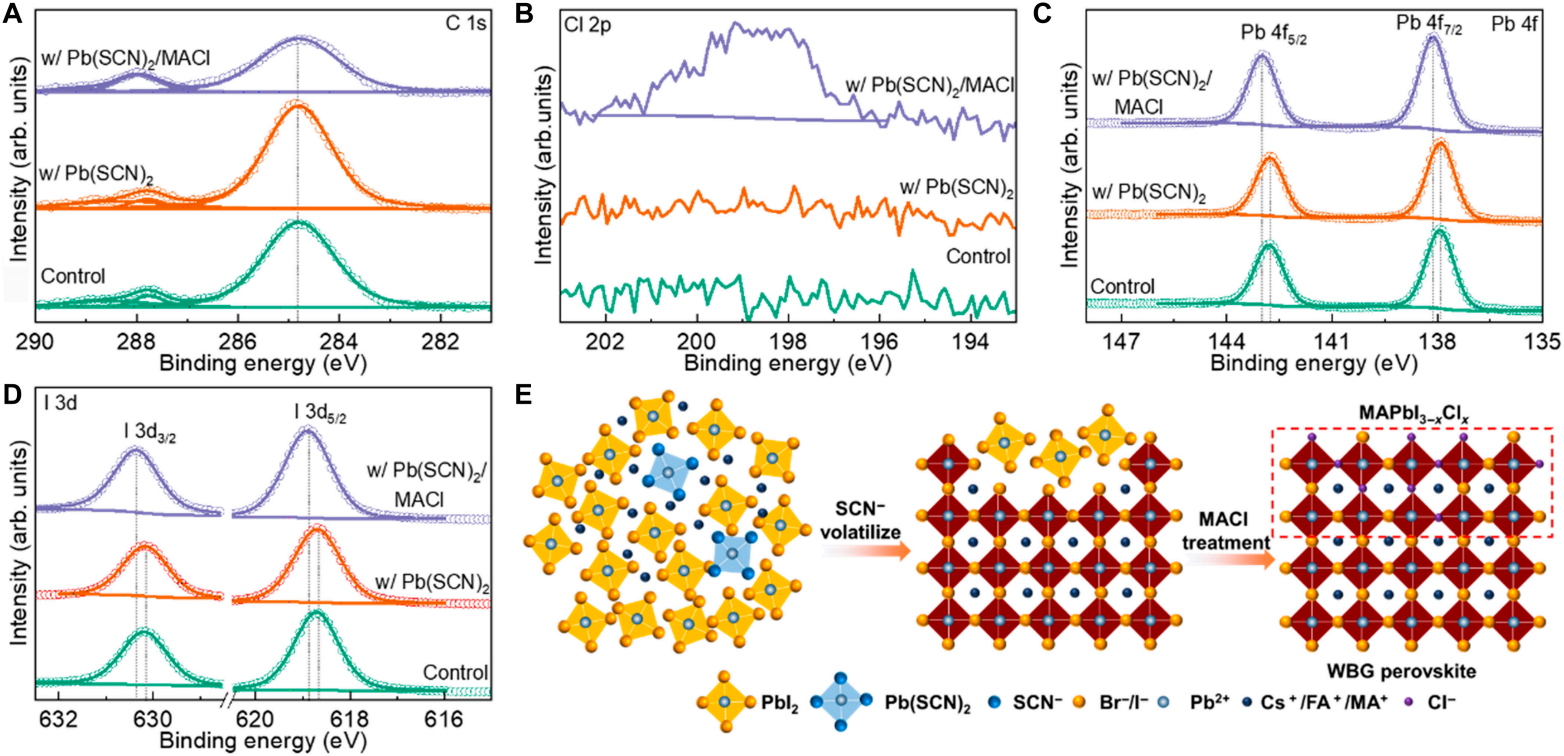
XPS core-level spectra for (A) C 1s, (B) Cl 2p, (C) Pb 4f, and (D) I 3d of control WBG perovskite film, the samples with Pb(SCN)_2_ additive, and MACl posttreatment. (E) Schematic of component variation in the WBG perovskite film with Pb(SCN)_2_ additive and MACl posttreatment.

The FA_0.65_MA_0.20_Cs_0.15_Pb(I_0.8_Br_0.2_)_3_ has an optimal bandgap of 1.68 eV for perovskite/silicon tandem devices to achieve high efficiency [14,26]. The low ratio of bromide and iodide is conducive to suppressing halide phase segregation [38]. However, with the increased content of cesium, the crystal quality of perovskite films and *V*_OC_ of WBG PSCs decreases gradually. Thus, we employed the Pb(SCN)_2_ additive to enhance crystallization and MACl posttreatment to remove excessive PbI_2_. As shown in Fig. 1A, all x-ray photoelectron spectroscopy (XPS) spectra were calibrated using 284.8 eV of C 1s as a reference point [20]. S 2p was not detected in any of the samples (Fig. S1), indicating that SCN^−^ was volatilized during the annealing process. The perovskite film with both Pb(SCN)_2_ additive and MACl posttreatment shows the presence of Cl 2p (Fig. 1B), suggesting that the chlorine ions are diffused into the crystal lattice. Furthermore, the Pb 4f and I 3d peaks show no shift in the sample with Pb(SCN)_2_ additive, while those with MACl posttreatment exhibited a shift toward higher binding energy (Fig. 1C and D), indicating changes in the local chemical states of the sample. As illustrated in Fig. 1E, the volatilization of SCN^−^ ions during annealing resulted in the formation of excessive PbI_2_ in perovskite films with Pb(SCN)_2_ additive. The excessive PbI_2_ then reacted with MACl during posttreatment, forming MAPbI_3−*x*_Cl*_x_* perovskite and WBG/MAPbI_3–*x*_Cl*_x_* GHJ on the surface. The depth analysis was employed to confirm the distribution of GHJ after Ar^+^ etching at different times. As shown in Fig. S2, the chloride ions can be detected after etching 30 s, whereas they disappeared after etching 60 s. Moreover, the Pb 4f peak shifted to the higher binding energy after etching, whereas the I 3d peak shifted to a lower value. This indicates that the GHJ is formed near the upper surface.

**Fig. 2.**
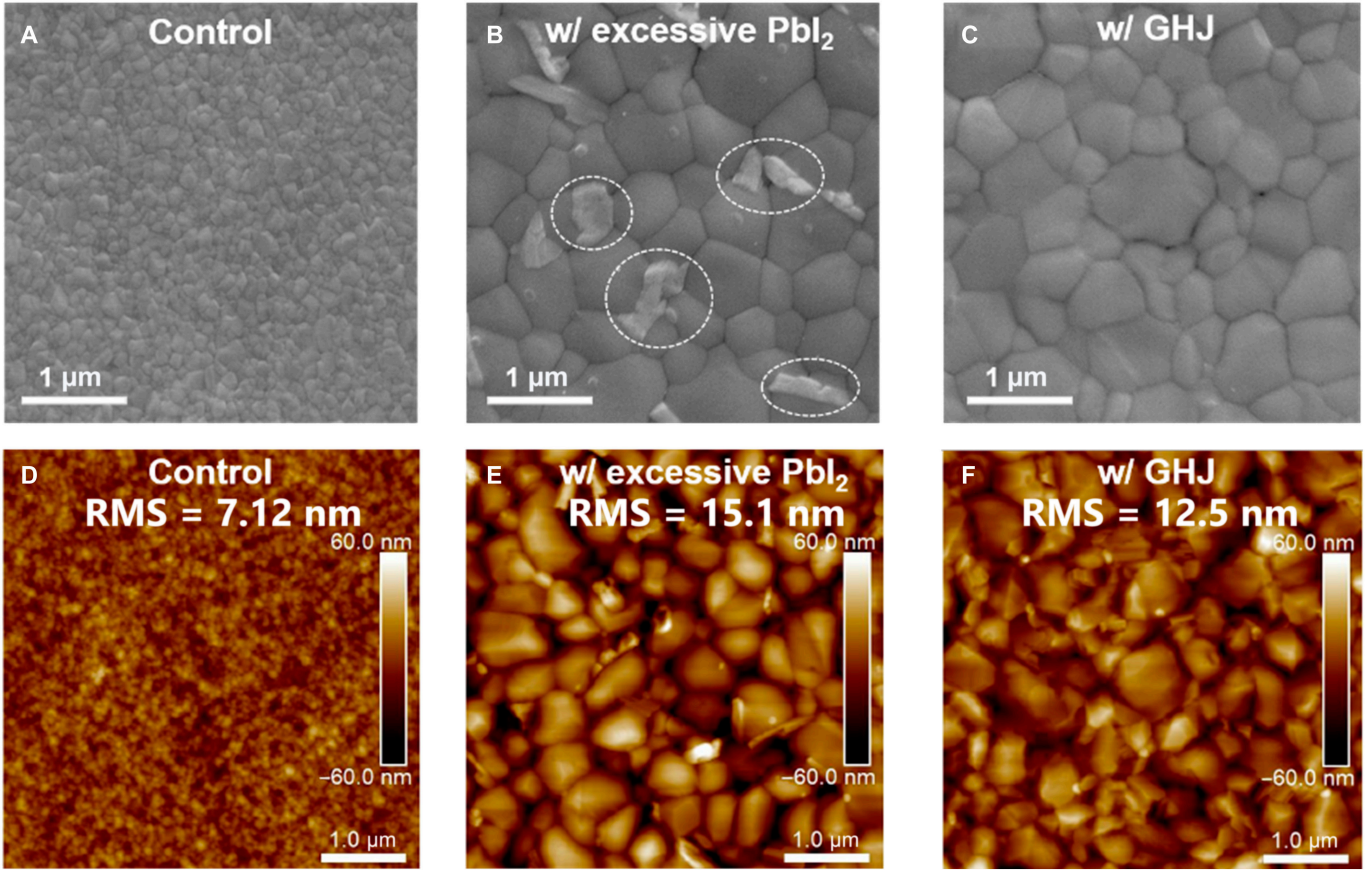
SEM images of (A) the control sample, and WBG films with (B) excessive PbI_2_ and (C) GHJ, respectively. AFM images of (D) the control sample, and WBG films with (E) excessive PbI_2_ and (F) GHJ, respectively.

Figure 2A to C provides scanning electron microscope (SEM) images, in which the grain is visibly larger after introducing Pb(SCN)_2_ additive, indicating improved crystallinity and a reduction in defects. The statistical analysis of grain size, as presented in Fig. S3, indicates that the average value increases from 198.8 to 747.4 nm after adding Pb(SCN)_2_. The excessive PbI_2_ is formed at grain boundaries, which can be removed by MACl posttreatment [39,40]. However, MACl posttreatment has a negligible effect on crystallinity. As shown in Fig. S4, residual PbI_2_ is observed in the samples containing Pb(SCN)_2_ additive, even after washing with isopropyl alcohol (IPA), suggesting that the excessive PbI_2_ has indeed reacted with MACl. As the atomic force microscope images are presented in Fig. 2E to G, the Pb(SCN)_2_ additive increased surface roughness owing to coarsened grains and excessive PbI_2_. However, MACl posttreatment resulted in a reduction in surface roughness, indicating that excessive PbI_2_ was removed. Therefore, the synergistic of Pb(SCN)_2_ additive and MACl posttreatment can improve the crystallization of WBG films.

**Fig. 3.**
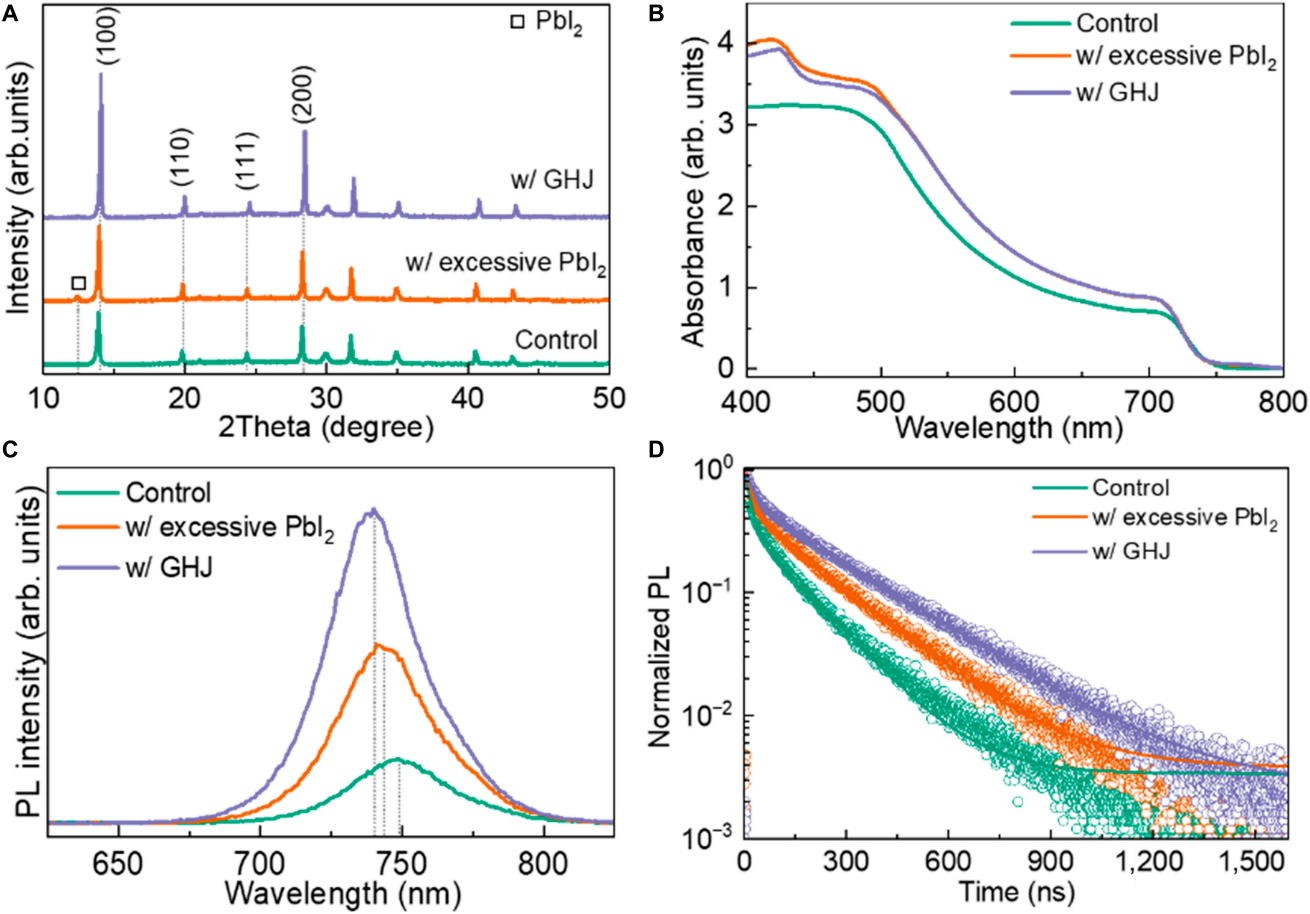
(A) XRD patterns, (B) UV-vis absorption spectra, (C) PL spectra, and (D) TRPL curves of the control sample, and WBG films with excessive PbI_2_ and GHJ, respectively.

X-ray diffraction (XRD) was employed to perform the crystalline structure of the WBG films with Pb(SCN)_2_ additive and subsequent treatment with MACl [41]. Two distinctive diffraction peaks are seen at 14.06° and 28.19°, which correspond to the (100) and (200) crystal planes of the α-phase, as presented in Fig. 3A [36,42]. Additionally, a new peak appears at 12.38° corresponding to PbI_2_, indicating that Pb(SCN)_2_ additive formed excessive PbI_2_ on the surface [43]. The intensity of (100) and (200) planes is enhanced in the samples with the Pb(SCN)_2_ additive and MACl posttreatment [44]. Meantime, the diffraction peaks shifted to a higher degree (Fig. S5), resulting from the excessive PbI_2_ and smaller chloride doped in the crystal. As shown in Fig. S6, the diffraction intensity of PbI_2_ and (100) plane is enhanced with the concentration of Pb(SCN)_2_ additive increasing. Comprehensively, the optimal concentration of Pb(SCN)_2_ additive is 2%. As presented in Fig. S7A, the optimal concentration of MACl solution is 3 mg/ml, as demonstrated by the highest diffraction intensity and the disappearance of the PbI_2_ peak. Moreover, Fig. S7B illustrates that the (100) peak shifts to a higher degree as the concentration of MACl increases. This demonstrates that the compressive strain is formed on the surface owing to the smaller crystal lattice of MAPbI_3–*x*_Cl*_x_* than WBG perovskite [45]. Based on these results, we speculate that chloride ions are incorporated into the crystal lattice to form GHJ. To further investigate this effect, we prepared samples containing 2% Pb(SCN)_2_ additive and 3 mg/ml MACl. As presented in Fig. 3B, a distinct absorption onset is at around 750 nm in ultraviolet (UV)-visible (vis) absorption spectra. The absorption edge blue-shifted in the perovskite films with Pb(SCN)_2_ additive and MACl posttreatment, indicating the presence of excessive PbI_2_ and GHJ [39,46]. In addition, the absorbance is also enhanced owing to the improved crystallization after adding Pb(SCN)_2_. Tauc curves were calculated using absorption spectra in Fig. S8. In comparison with the control sample, the optical bandgap of WBG films with excessive PbI_2_ and GHJ enhanced from 1.686 to 1.696 and 1.698 eV, respectively. The steady-state photoluminescence (PL) spectra were measured using the samples prepared on an insulated substrate and covered by poly (methyl methacrylate) (PMMA). The control sample exhibits an emission peak located at around 749 nm, whereas the perovskite films with excessive PbI_2_ and GHJ exhibit blue shift and enhanced intensity resulting from the suppression of nonradiative recombination [19,47]. Moreover, the PL spectra were measured from both the glass and PMMA sides to perform the difference between bulk and surface in Fig. S9. The perovskite films with GHJ exhibit a blue shift measured from the PMMA side, indicating that the GHJ is mainly located on the surface. The PL spectra were recorded after light soaking for 2 h in Fig. S10, where the sample with GHJ did not show halide segregation under continuous illumination in comparison with the control one. This demonstrates that the compressive strain resulted in higher barrier of ion migration, suggesting that the GHJ can improve light stability and enhance operational stability [45,48]. Moreover, time-resolved photoluminescence (TRPL) is utilized to determine the carrier lifetime [26]. As presented in Fig. 3D and Table S1, the average lifetime was increased from 92.31 ns to 173.91 and 231.12 ns in the WBG films with excessive PbI_2_ and GHJ, indicating enhanced carrier lifetime resulting from improved crystallization and passivated defects. Next, we further investigate the effect of GHJ on WBG films through electronic characterizations.

**Fig. 4.**
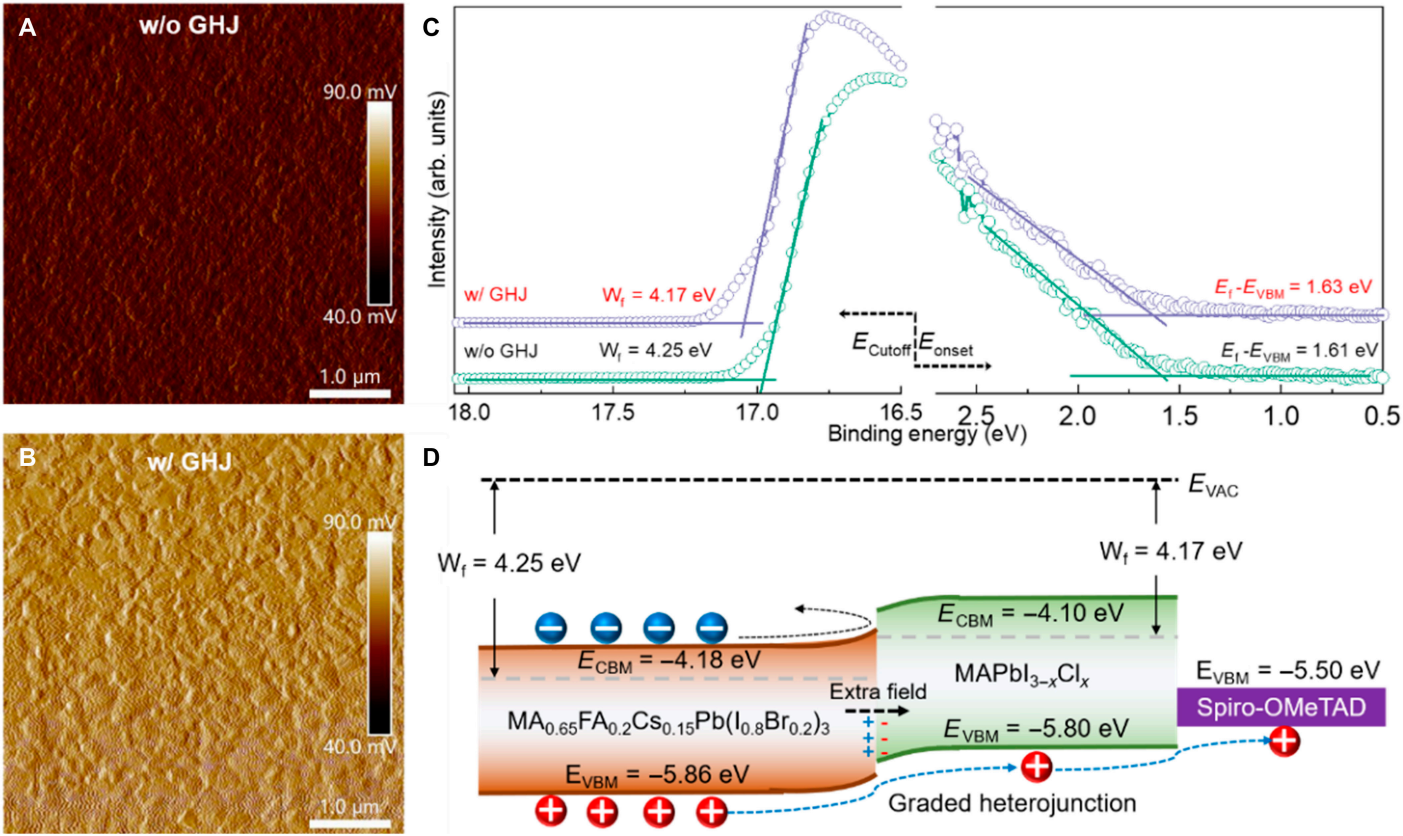
KPFM images of WBG perovskite film (A) without and (B) with GHJ, respectively. (C) UV photoelectron spectroscopy spectra of WBG perovskite film without and with GHJ. (D) The schematic energy band diagrams of WBG perovskite film with GHJ.

To investigate the surface variation, the contact potential difference (CPD) is determined by Kelvin probe force microscopy (KPFM). As presented in Fig. 4A and B, the CPD of perovskite film with GHJ is larger than the sample without GHJ, indicating a shift in the Fermi level upwards, which is beneficial for facilitating charge separation and carrier extraction [49–51]. In addition, the valence band and work function (*W*_f_) are obtained by UV photoelectron spectroscopy [51,52]. As shown in Fig. 4C, the *W*_f_ is observed to decrease from 4.25 to 4.17 eV owing to the effect of GHJ, consisting well of the CPD change measured by KPFM. Furthermore, the valence band maximum (*E*_VBM_) and conduction band minimum (*E*_CBM_) were determined using the optical bandgap obtained from absorption. The energy level alignment is presented in Fig. 4D. An extra built-in electrical field is formed at GHJ to facilitate hole extraction and reduce charge recombination [34,53]. These results indicate that GHJ is beneficial for improving the electrical performance of WBG perovskite films.

**Fig. 5.**
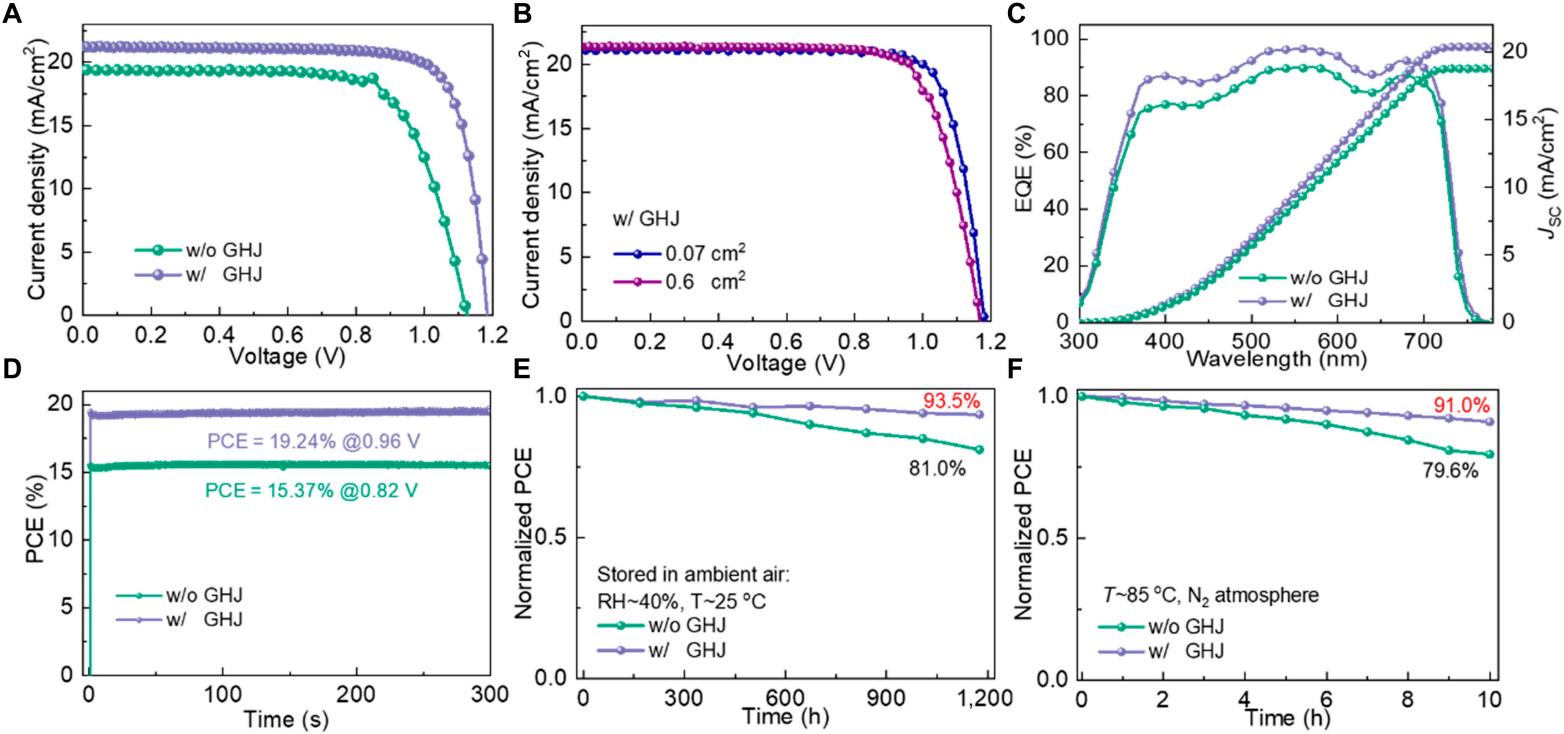
(A) Light *J*-*V* curves of WBG PSCs without and with GHJ. (B) Light *J*-*V* curves of WBG PSCs with GHJ in active areas of 0.07 and 0.6 cm^2^. (C) EQE spectra and integrated current of WBG PSCs without and with GHJ. (D) Steady output efficiency at MPP, (E) long-term storage stability in ambient, and (F) thermal stability at 85 °C of WBG PSCs without and with GHJ, respectively.

We fabricated the PSCs with a configuration of ITO/SnO_2_/WBG perovskite/Spiro-OMeTAD/Ag. Figure 5A presents the current density-voltage (*J*-*V*) curves of WBG PSCs without and with GHJ. The optimal WBG PSC with GHJ exhibited a high PCE of 20.30% with a *V*_OC_ of 1.185 V and fill factor (FF) of 0.805, whereas the control one displayed a low efficiency of 15.48% with a *V*_OC_ of 1.101 V and FF of 0.726, as shown in Table. Moreover, the short-circuit current density (*J*_SC_) is also enhanced from 19.37 to 21.28 mA/cm^2^. Additionally, the WBG PSC with GHJ demonstrated a lower hysteresis index than the one without GHJ (Fig. S11 and Table S2), indicating the suppression of defects and ions migration. As shown in Fig. 5B, the device with an area of 0.6 cm^2^ displayed a PCE of 18.88%, which is slightly lower than the device with an area of 0.07 cm^2^ of 19.89% (Table S3). The large-area device with 0.6 cm^2^ further demonstrated the uniformity of GHJ on WBG perovskite films. To verify reproducibility, the photovoltaic parameters statistics of 20 independent devices are presented in Fig. S12. This also demonstrates the marked enhancement of *V*_OC_, FF, and *J*_SC_ in the device with GHJ, resulting from improved crystallinity, passivated defects, and beneficial energy level alignment.

**Table.** Photovoltaic parameters for WBG PSCs without and with GHJ, respectively.

Samples	*J*_SC_ (mA/cm^2^)	*V*_OC_ (V)	FF	PCE (%)
w/o GHJ	19.37	1.101	0.726	15.48
w/ GHJ	21.28	1.185	0.805	20.30

The external quantum efficiency (EQE) is presented in Fig. 5C, where the response of the PSC with GHJ is higher than the control one owing to its fast carrier extraction. Thus, the integrated current densities match well with the *J*_SC_ in *J*-*V* results. Meantime, the cutoff is approximately 750 nm, consisting well with the absorption. Additionally, the steady output efficiency was recorded at the maximum power point (MPP) for 300 s, as presented in Fig. 5D. The WBG PSC with GHJ displays a steady-output PCE of 19.24%, indicating its excellent operational stability. To further investigate the illumination stability of the WBG PSCs, periodic photo-responses were measured in ambient air with a relative humidity (RH) of ~50%. The current was recorded under illumination with an on/off-modulated 405-nm laser, as shown in Fig. S13. The current remained almost undiminished for 19.5 h under alternating illumination and darkness conditions, indicating excellent operational stability exhibited in the WBG PSCs with GHJ in ambient air. The storage stability was tracked in ambient air with RH of approximately 40% and room temperature. As shown in Fig. 5E, the unencapsulated device with GHJ can maintain 93.5% of its initial efficiency after 1,200 hours of storage, but the control device only retained 81.0%. Moreover, the device with GHJ retained 91.0% of its initial efficiency after heating for 8 h, but the control sample decreased to 73.6%, as shown in Fig. 5F. This demonstrates that the GHJ is employed to improve the stability of WBG PSC owing to compressive strain on the surface. Overall, the WBG PSC with GHJ displays outstanding photovoltaic performance and stability in comparison with the control one.

**Fig. 6.**
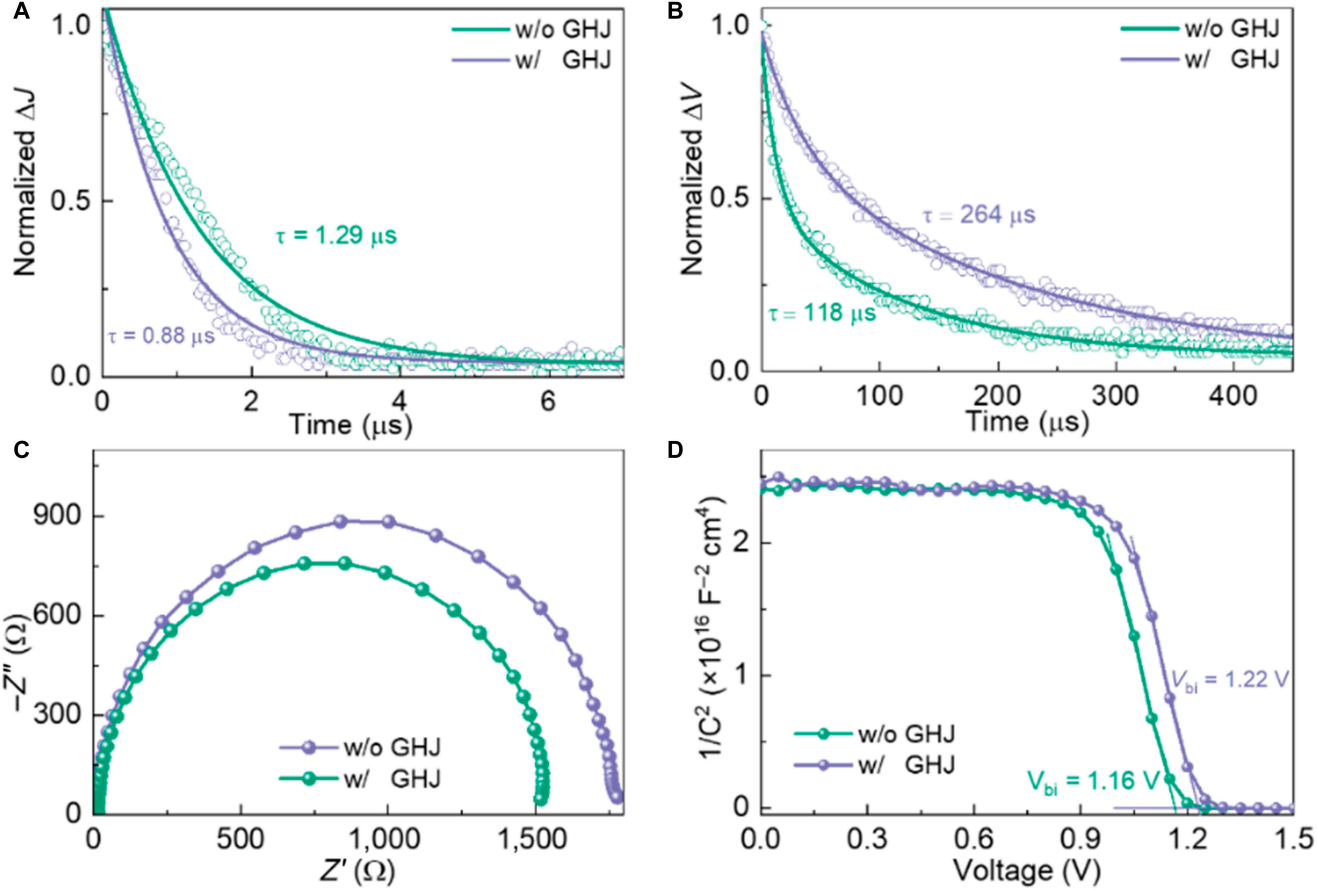
(A) Transient photocurrent, (B) transient photovoltage, (C) Nyquist, and (D) Motto–Schottky curves of WBG PSCs without and with GHJ, respectively.

To better understand the reason for GHJ on performance enhancement, several analyses were employed to examine the carrier dynamics. The space charge limited current is used to evaluate the trap-state density (*n*_t_), which is proportional to the trap-filled limit voltage (*V*_TFL_) [54,55]. As presented in Fig. S14, the *V*_TFL_ is decreased from 0.44 to 0.31 V, suggesting that defects are passivated in PSC with GHJ. The carrier-extraction and carrier-recombination lifetime was measured by transient photocurrent and transient photovoltage, respectively [56,57]. As shown in Fig. 6A and B, the WBG PSC with GHJ demonstrated a lower carrier-extraction lifetime (0.88 μs) than the control one (1.29 μs), indicating that the GHJ can facilitate carrier extraction at the interface. Meantime, the device with GHJ enhanced the carrier-recombination lifetime from 118 to 264 μs, suggesting that the defects on perovskite films are passivated. Moreover, Nyquist plots were obtained by ac impedance and fitted with an equivalent circuit (Fig. S15). As presented in Fig. 6C, the larger recombination resistance (1,775 Ω) exhibits in the device with GHJ than in the control one (1,522 Ω), resulting from the passivated defects on the surface [30]. In addition, the series resistance is reduced from 17 to 15 Ω owing to the improved crystallization, leading to the high *J*_SC_ of devices with GHJ. Furthermore, the Motto–Schottky plots were used to estimate the built-in potential (*V*_bi_) by capacitance-voltage (*C*-*V*) measurement [42]. As demonstrated in Fig. 6D, the *V*_bi_ was increased from 1.16 to 1.22 V because of the suppressed nonradiative recombination and improved energy level alignment. The larger *V*_bi_ is conducive to increasing the force of carrier extraction and stretching depletion region, further enhancing the *V*_OC_ of PSC [28,58].

**Fig. 7.**
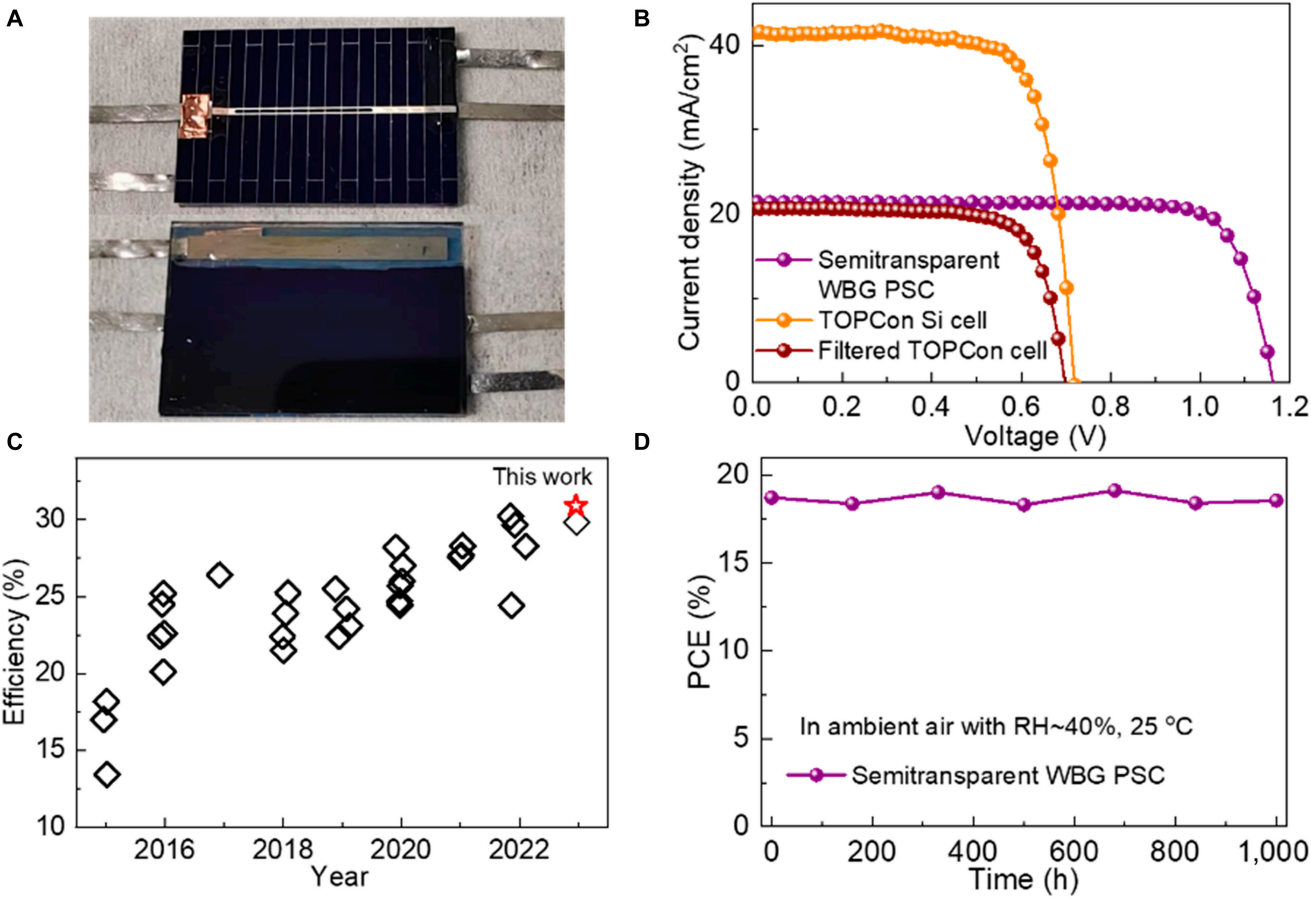
(A) Photograph of the fabricated 4-T WBG perovskite/TOPCon silicon tandem device. (B) *J*-*V* curves of semitransparent WBG PSC as well as TOPCon silicon solar cells before and after filtering with the semitransparent device. (C) Summary of the state-of-the-art efficiencies of 4-T perovskite-silicon tandem solar cells. (D) PCE variation of semitransparent WBG PSC after storage in ambient air.

The 4-T tandem solar cells were fabricated by combining semitransparent WBG PSCs with TOPCon silicon solar cells, as presented in Fig. 7A. A thin layer of molybdenum oxide (MoO_x_) was deposited on Spiro-OMeTAD to protect it during the preparation of the indium-zinc-oxide transparent electrode by sputtering. The *J*-*V* curves were independently measured for semitransparent WBG PSC and TOPCon silicon solar cells. As presented in Fig. 7B and Table S4, the semitransparent WBG PSC exhibited an efficiency of 20.13%. In addition, the TOPCon silicon solar cell displayed a PCE of 22.50%, which decreased to 10.78% after filtering with semitransparent WBG PSC. As shown in Fig. S16, a semitransparent WBG device with an area of 4.5 × 4.5 cm^2^ was used as a filter during to test silicon solar cells. Consequently, the 4-T tandem device eventually achieved a recorded PCE of 30.91%. Figure 7C and Table S5 summarize the state-of-the-art efficiencies of 4-T perovskite-silicon tandem solar cells, which indicate obviously that the PCE achieved herein represents the highest level among them. To demonstrate the reliability of *J*-*V* results, we also measured the EQE response of the semitransparent WBG PSC, as depicted in Fig. S17. The integrated current is 20.68 mA/cm^2^, close to the J_SC_ acquired in the *J*-*V* results. Now, the operational lifetime of silicon solar cells is up to 25 years. Thus, the reliability of the tandem device depends on the perovskite material. As demonstrated in Fig. 7D, the efficiency of semitransparent WBG PSC remained at 18.55% after storage for 1,000 h in ambient air with an RH of 40%. The compact indium-zinc-oxide electrode effectively prevented humidity erosion, and the perovskite film was improved by GHJ, resulting in excellent stability of the semitransparent solar cells. These outstanding results suggest that GHJ has the potential to improve WBG PSCs while also enabling the development of highly efficient and stable perovskite/silicon tandem solar cells.

## Discussion

In summary, we employed the conventional additive of Pb(SCN)_2_ and MACl posttreatment to construct GHJ on the surface of WBG PSCs. The Pb(SCN)_2_ additive improved crystallinity, and formed excessive PbI_2_, while MACl reacted with excessive PbI_2_ and transformed it into MAPbI_3−*x*_Cl*_x_* perovskite. This results in the formation of GHJ and compressive strain, which were beneficial in passivating defects, suppressing ion migration, and facilitating carrier extraction. Consequently, the WBG PSC with GHJ achieved a remarkable PCE of 20.30%, with a high *V*_OC_ of 1.185 V, and retained 93.5% of the initial value after storage in ambient air with an RH of 40% for 1,200 h. Moreover, the 4-T WBG perovskite/TOPCon silicon tandem device shows a recorded efficiency of 30.91% and excellent stability. These findings have important implications for the fabrication of highly efficient and stable perovskite/silicon tandem solar cells.

## Materials and Methods

The materials and methods can be found in the Supplementary Materials.

## Data Availability

All data are available in the main text or the Supplementary Materials.

## Supplementary Materials

Supplementary 1 Materials and Methods. Fig. S1. XPS core-level spectra for S 2p of control WBG perovskite film, the samples with Pb(SCN)_2_ additive, and MACl post-treatment. Fig. S2. (A) XPS spectra of WBG perovskite film with GHJ after etching with 0, 30, and 60 s. Core-level (B) Cl 2p, (C) Pb 4f, and (D) I 3d XPS spectra of WBG perovskite film with GHJ after etching with 0, 30, and 60 s, respectively. Fig. S3. The statistical grain size of (A) control sample as well as WBG films with (B) excessive PbI_2_ and (C) GHJ, respectively. Fig. S4. SEM images of WBG films with excessive PbI_2_ (A) before and (B) after IPA washing, respectively. Fig. S5. Magnified (100) and (200) XRD peaks of the control sample as well as WBG films with excessive PbI_2_ and GHJ, respectively. Fig. S6. XRD patterns of control sample as well as WBG films with 1%, 2%, 3% Pb(SCN)_2_ additive. Fig. S7. (A) XRD patterns and (B) magnified (100) XRD peak of WBG films with 2% Pb(SCN)_2_ additive after treating with 0, 1, 3, and 5 mg/mL MACl/IPA solution. Fig. S8. Tauc curves of (A) control sample as well as WBG films with (B) excessive PbI_2_ and (C) GHJ, respectively. Fig. S9. PL spectra measured from PMMA and glass sides, respectively. Fig. S10. PL spectra of WBG films (A) without and (B) with GHJ measured after light soaking for 0, 1, and 2 h, respectively. Fig. S11. *J*-*V* curves measured by forward and reverse scanning, respectively. Fig. S12. Statistical (A) *V*_OC_, (B) FF, (C) J_SC_, and (D) PCE of 20 independent WBG PSCs without and with GHJ, respectively. Fig. S13. Response properties evaluated with an on/off-modulated 405 nm laser (40 mW cm^−2^) for the typical WBG PSC with GHJ recorded in ambient air. Fig. S14. SCLC curves of WBG films (A) without and (B) with GHJ, respectively. Fig. S15. Equivalent circuit model for fitting the Nyquist plots. Fig. S16. Photograph of large-area semitransparent WBG device used as a filter. Fig. S17. EQE and integrated current of semitransparent WBG PSC. Table S1. Fitting parameters for the TRPL decay of the control sample as well as WBG films with excessive PbI_2_ and GHJ, respectively. Table S2. Photovoltaic parameters for WBG PSCs without and with GHJ measured by forward and reverse scanning, respectively. Table S3. Photovoltaic parameters for WBG PSCs with GHJ in an area of 0.07 and 0.6 cm^2^. Table S4. Photovoltaic parameters for semitransparent WBG PSC as well as TOPCon silicon solar cells before and after filtering with the semitransparent device. Table S5. A summary of the state-of-the-art efficiencies of semi-transparent PSCs and 4-T perovskite/silicon tandem devices.
