# Supplementary material for: Graded Heterojunction Improves Wide-Bandgap Perovskite for Highly Efficient 4-Terminal Perovskite/Silicon Tandem Solar Cells
Source: Research (Wash D C). 2023 Jul 17;6:0196. doi: 10.34133/research.0196 (PMC10351391; doi:10.34133/research.0196)
Supplement: Supplementary 1 — Materials and Methods. Fig. S1. XPS core-level spectra for S 2p of control WBG perovskite film, the samples with Pb(SCN)2 additive, and MACl post-treatment. Fig. S2. (A) XPS spectra of WBG perovskite film with GHJ after etching with 0, 30, and 60 s. Core-level (B) Cl 2p, (C) Pb 4f, and (D) I 3d XPS spectra of WBG perovskite film with GHJ after etching with 0, 30, and 60 s, respectively. Fig. S3. The statistical grain size of (A) control sample as well as WBG films with (B) excessive PbI2 and (C) GHJ, respectively. Fig. S4. SEM images of WBG films with excessive PbI2 (A) before and (B) after IPA washing, respectively. Fig. S5. Magnified (100) and (200) XRD peaks of the control sample as well as WBG films with excessive PbI2 and GHJ, respectively. Fig. S6. XRD patterns of control sample as well as WBG films with 1%, 2%, 3% Pb(SCN)2 additive. Fig. S7. (A) XRD patterns and (B) magnified (100) XRD peak of WBG films with 2% Pb(SCN)2 additive after treating with 0, 1, 3, and 5 mg/mL MACl/IPA solution. Fig. S8. Tauc curves of (A) control sample as well as WBG films with (B) excessive PbI2 and (C) GHJ, respectively. Fig. S9. PL spectra measured from PMMA and glass sides, respectively. Fig. S10. PL spectra of WBG films (A) without and (B) with GHJ measured after light soaking for 0, 1, and 2 h, respectively. Fig. S11. J-V curves measured by forward and reverse scanning, respectively. Fig. S12. Statistical (A) VOC, (B) FF, (C) JSC, and (D) PCE of 20 independent WBG PSCs without and with GHJ, respectively. Fig. S13. Response properties evaluated with an on/off-modulated 405 nm laser (40 mW cm−2) for the typical WBG PSC with GHJ recorded in ambient air. Fig. S14. SCLC curves of WBG films (A) without and (B) with GHJ, respectively. Fig. S15. Equivalent circuit model for fitting the Nyquist plots. Fig. S16. Photograph of large-area semitransparent WBG device used as a filter. Fig. S17. EQE and integrated current of semitransparent WBG PSC. Table S1. Fitting parameters for the TRPL [file research.0196.f1.docx]

# Supplementary Materials

**Graded heterojunction improves wide-bandgap perovskite for highly efficient 4-terminal perovskite/silicon tandem solar cells**

*Wenming Chai,^a,‡^ Lindong Li,^a,‡^ Weidong Zhu,^a,b,*^ Dazheng Chen,^a,b^ Long Zhou,^a,b^ He Xi,^a^ Jincheng Zhang,^a^ Chunfu Zhang,^a,*^ and Yue Hao ^a^*

^a^State Key Discipline Laboratory of Wide Band Gap Semiconductor Technology & Shaanxi Joint Key Laboratory of Graphene, School of Microelectronics, Xidian University, Xi’an, Shaanxi 710071, China.

^b^Xi'an Baoxin Solar Technology Co., Ltd., Xi'an, 710071, China.

^‡^The authors contribute equally to the work.

*E-mail: wdzhu@xidian.edu.cn; cfzhang@xidian.edu.cn

**Experimental section**

*Materials:* Formamidinium iodide (FAI, 99.99%), methylammonium bromide (MABr, 99.99%), tris(2-(1H-pyrazol-1-yl)-4-tert-butylpyridine)-cobalt(III) tris (bis (trifluoromethylsulfonyl) imide) (FK209 Co(III) TFSI, 98%) were purchased from Greatcell Solar. Lead iodide (PbI_2_, 99.999%), lead bromide (PbBr_2_, 99.999%), cesium iodide (CsI, 99.998%), SnO_2_ aqueous solution (15% in H_2_O colloidal dispersion liquid) were purchased from Alfa Aesar. Lead thiocyanate (Pb(SCN)_2_, 99.5%), bis (trifluoromethane) sulfonimide lithium salt (Li-TFSI, 99.95%), N,N-dimethylformamide (DMF, 99.8%), molybdenum Oxide (MoO_x_, 99.97%), chlorobenzene (CB, 99.8%), isopropanol (IPA, 99.5%), 4-tert-butylpyridine (tBP, 98%) were purchased from Sigma-Aldrich. N-methyl-2-pyrrolidone (NMP, 99%), and acetonitrile (99.9%) were purchased from J&K Scientific. Methylamine chloride (MACl, 99%) and 2,2’,7,7’-tetrakis (N,N-di-p-methoxyphenylamine)-9,9 spirobifluorene (Spiro-OMeTAD, 99.8%) were purchased from Xi’an Polymer Light Technology Corp. The Indium Zinc Oxide (IZO) target was purchased from ZhongNuo Advanced Material Technology Co. TOPCon silicon cells were purchased from Qinghai Huanghe Hydropower Development.

*Precursor preparation:* Wide-bandgap perovskite precursor solutions were prepared by dissolving 145.3 mg FAI, 29.1 mg MABr, 479.4 mg PbI_2_, 50.7 mg CsI, 95.4 mg PbBr_2_ in 1.0 mL mixed solvent of DMF and NMP (DMF: NMP= 4:1). For the device with GHJ, 2% Pb(SCN)_2_ was added in the precursor solution. MACl solution was prepared with a concentration of 3 mg mL^-1^ in IPA solvent. The SnO_2_ precursor solution was obtained by diluting a 15 wt% aqueous colloidal dispersion of SnO_2_ nanoparticles in deionized (DI) water (SnO_2_ aqueous solution: DI water = 1:2, volume ratio). The spiro-OMeTAD solution contained 72.3 mg spiro-OMeTAD dissolved in 1.0 mL CB with the additives 16.5 μL Li-TFSI (520 mg mL^-1^ in acetonitrile), 15.0 μL FK209 Co(Ⅲ)-TFSI (375 mg mL^-1^ in acetonitrile) and 28.5 μL tBP. The solutions were stirred at room temperature for 3 h before use.

*Device fabrication:* The ITO/glass substrates were ultrasonically cleaned sequentially with Decon90, deionized water, acetone, and ethanol for 15 min, respectively. The ITO/glass substrates were dried by N_2_ and treated with Ozone for 30 min. A thin SnO_2_ ETL was deposited on the ITO substrate by spin-coating at 3000 rpm for 30 s, then annealed at 150℃ for 30 min. The perovskite absorber was deposited on the SnO_2_ ETL after Ozone treatment for 15 min using a two-step spin coating process: 1000 rpm for 5 s, and 4000 rpm for 50 s. 200 μL of CB acting as an anti-solvent was poured on the spinning substrate at 12 s of total time. The intermediate films were annealed at 100℃ for 10 min in ambient air to obtain the crystallized (FA_0.65_MA_0.20_Cs_0.15_)Pb(I_0.8_Br_0.2_)_3_ film. For the MACl post-treatment samples, 60 μL MACl solution was further spin-coated at 4000 rpm for 30 s, followed by annealing at 100 ℃ for 5 min. As HTL, spiro-OMeTAD was deposited on perovskite films by spin-coating at 4000 rpm for 45 s and then exposed to dry air for 24 h. Finally, a 100 nm thick silver was deposited by thermal evaporation through masks to define the active area to 0.07 cm^2^. For semitransparent devices, 30 nm MoO_x_ was thermally evaporated on spiro-OMeTAD as a buffer layer to eliminate the damage during the IZO sputtering procedure. And then an 80 nm IZO transparent electrode with a sheet resistance of 39.2 Ω sq^-1^ was deposited by the RF magnetron sputtering process. To increase the conductivity of the rear IZO, 100 nm Ag fingers were deposited by thermal evaporation. For the measurements of the bottom cells in a 4-T configuration, semitransparent perovskite filters with the same structure and optical properties enable photon energy to pass through the glass side to the bottom cell.

*Fabrication of TOPCon solar cells:* The TOPCon silicon solar cells features tunnel SiO_x_ passivated rear contact and a diffused poly-Si (n^+^). To reduce the front side surface recombination, a boron-doped p^+^ emitter was passivated by a stack of atomic layer deposited (ALD) [aluminum oxide](https://www.sciencedirect.com/topics/materials-science/aluminum-oxide) (Al_2_O_3_) layer and plasma-enhanced chemical vapor deposited (PECVD) [silicon nitride](https://www.sciencedirect.com/topics/materials-science/silicon-nitride) (SiN_x_) layer. Besides, it has been well established that silicon nitride (SiN_x_) layer also serves the purpose of anti-reflection coating (ARC) so that optical confinement inside the solar cell can be enlarged. The front side metallic contacts were realized by thermally evaporated Ag/Al seed layer and subsequent electroplating of the Ag layer. On the other hand, thermally evaporated Ag stack was used as rear side metallic contact. Besides, the small-area devices were prepared by cutting with a 532 nm laser at a frequency of 1000 Hz.

*Characterizations:* XPS and UPS spectra were obtained from X-ray photoelectron spectroscopy (Nexsa, Thermo Fisher). SEM images were acquired by a desktop SEM (Phenom Pro). XRD was measured by an x’pert3 powder X-ray diffractometer (PANalytical, Netherlands). UV-vis absorption spectra were obtained by a spectrophotometer (U-4100, Hitachi). Steady-state PL and TRPL were tested on a FluoTime 300 spectrometer (PicoQuant, German). Light J-V curves were recorded from 1.3 to -0.2 V with a step of 0.03 V/s by a Keithley 2450 source meter under a simulated AM 1.5Gillumination (100 mW cm^−2^), which was produced by an Oriel 92251A-1000 sunlight simulator. EQE was carried out on a 150 W xenon lamp (Oriel) equipped with a monochromator (Cornerstone 74004). SCLC was measured by a Keithley 2450 source meter in dark conditions. A.C. impedance and Motto-Schottoky were tested on an electrochemical workstation (CHI 660B) under dark conditions. The 1V forward bias was applied to the A.C. impedance measurements. TPC and TPV were recorded by an oscilloscope, in which the sampling resistor of 50 Ω or 1 MΩ was applied. The photocurrent decay was measured under a 532 nm pulse laser (1000 Hz, 3.2 ns). The photovoltage decay was carried out under a 405 nm pulse laser (50 Hz, 20 ms).


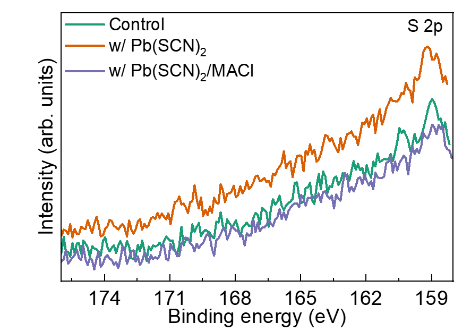


**Fig. S1.** XPS core-level spectra for S 2p of control WBG perovskite film, the samples with Pb(SCN)_2_ additive, and MACl post-treatment.


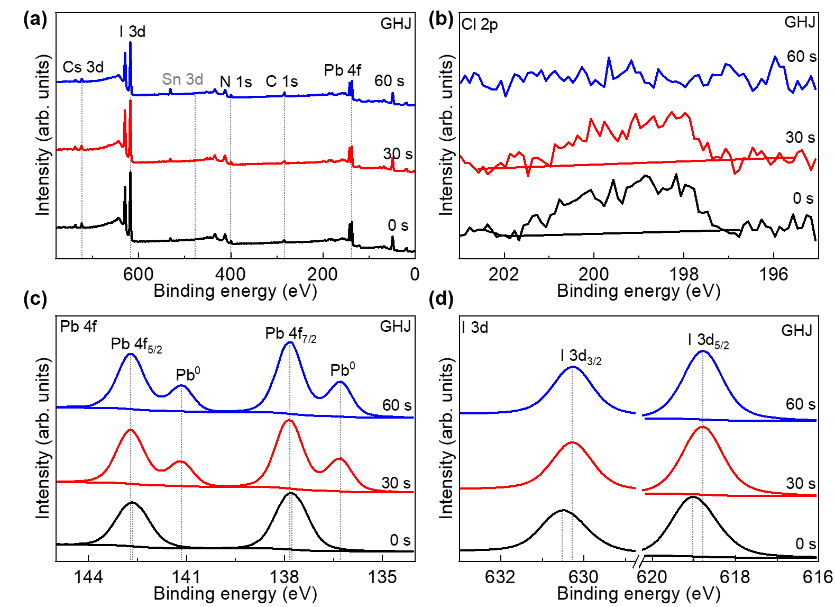


**Fig. S2** (a) XPS spectra of WBG perovskite film with GHJ after etching with 0, 30, and 60 s. Core-level (b) Cl 2p, (c) Pb 4f, and (d) I 3d XPS spectra of WBG perovskite film with GHJ after etching with 0, 30, and 60 s, respectively.


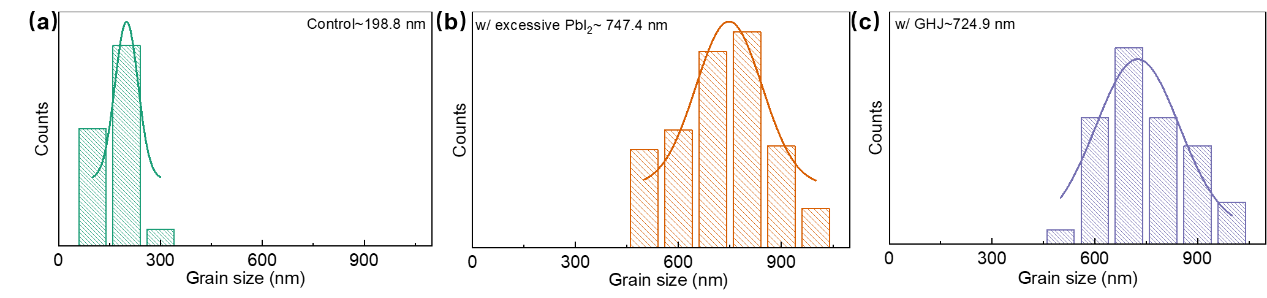


**Fig. S3.** The statistical grain size of (a) control sample as well as WBG films with (b) excessive PbI_2_ and (c) GHJ, respectively.


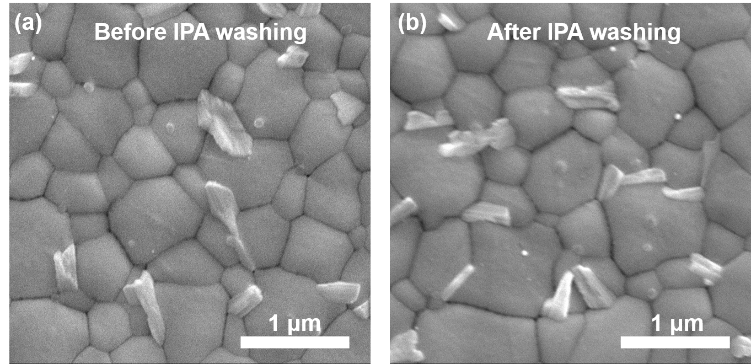


**Fig. S4.** SEM images of WBG films with excessive PbI_2_ (a) before and (b) after IPA washing, respectively.


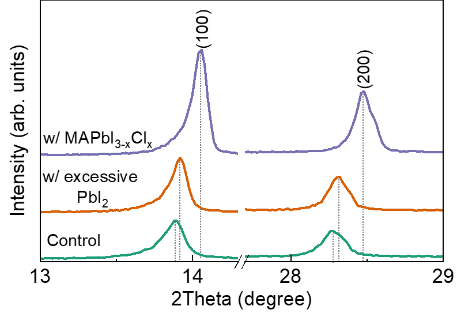


**Fig. S5.** Magnified (100) and (200) XRD peaks of the control sample as well as WBG films with excessive PbI_2_ and GHJ, respectively.


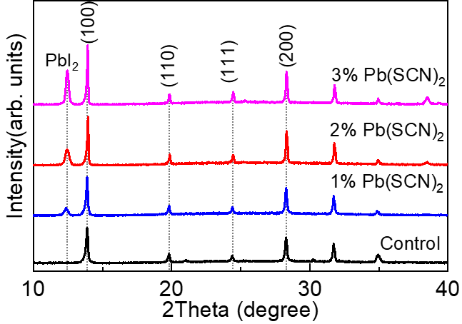


**Fig. S6.** XRD patterns of control sample as well as WBG films with 1%, 2%, 3% Pb(SCN)_2_ additive.


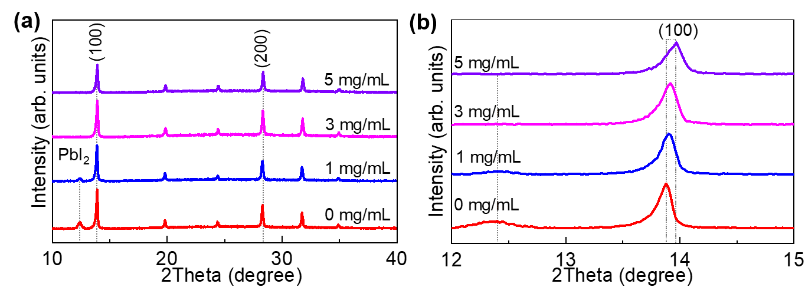


**Fig. S7.** (a) XRD patterns and (b) magnified (100) XRD peak of WBG films with 2% Pb(SCN)_2_ additive after treating with 0, 1, 3, and 5 mg/mL MACl/IPA solution.


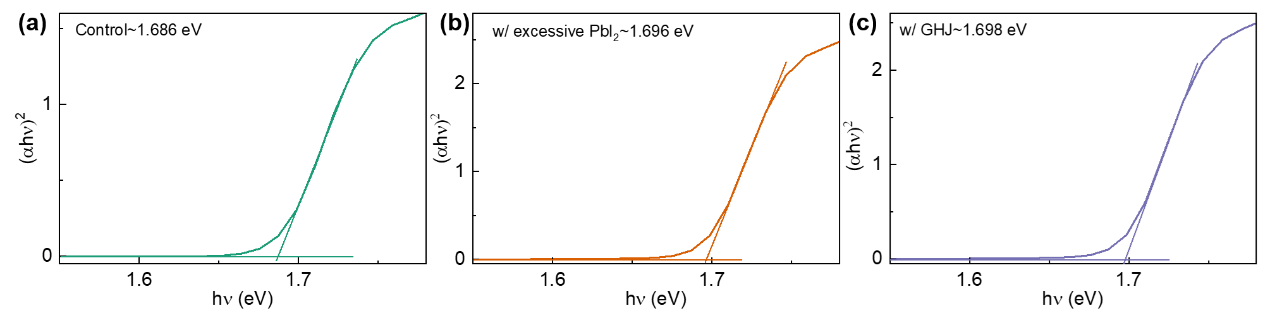


**Fig. S8.** Tauc curves of (a) control sample as well as WBG films with (b) excessive PbI_2_ and (c) GHJ, respectively.


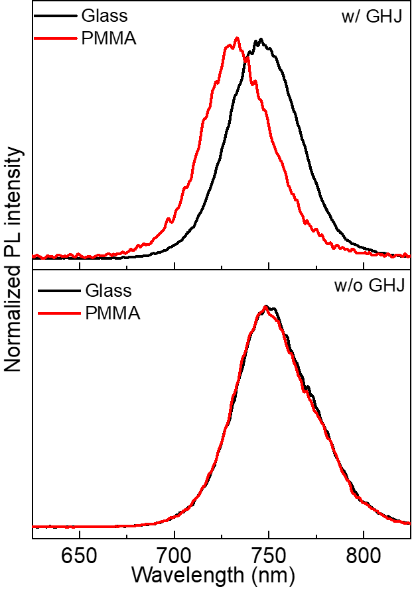


**Fig. S9.** PL spectra measured from PMMA and glass sides, respectively.


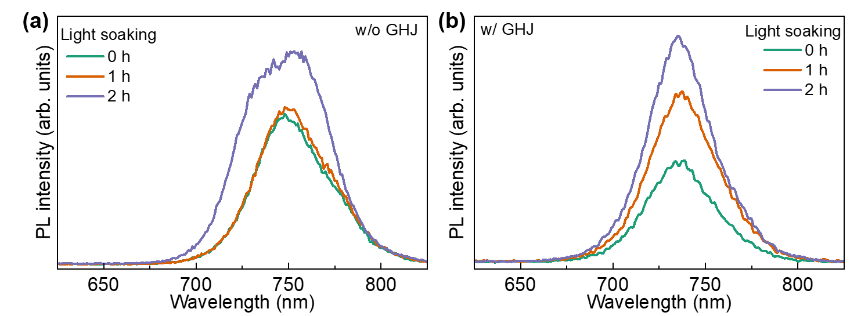


**Fig. S10.** PL spectra of WBG films (a) without and (b) with GHJ measured after light soaking for 0, 1, and 2 h, respectively.


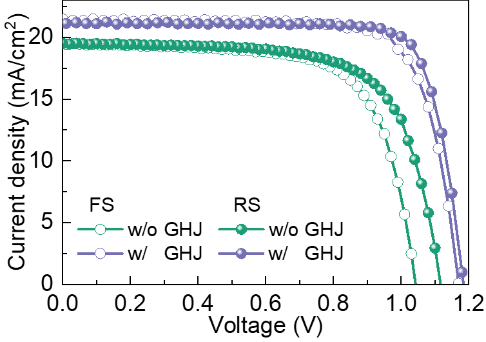


**Fig. S11.** J-V curves measured by forward and reverse scanning, respectively.


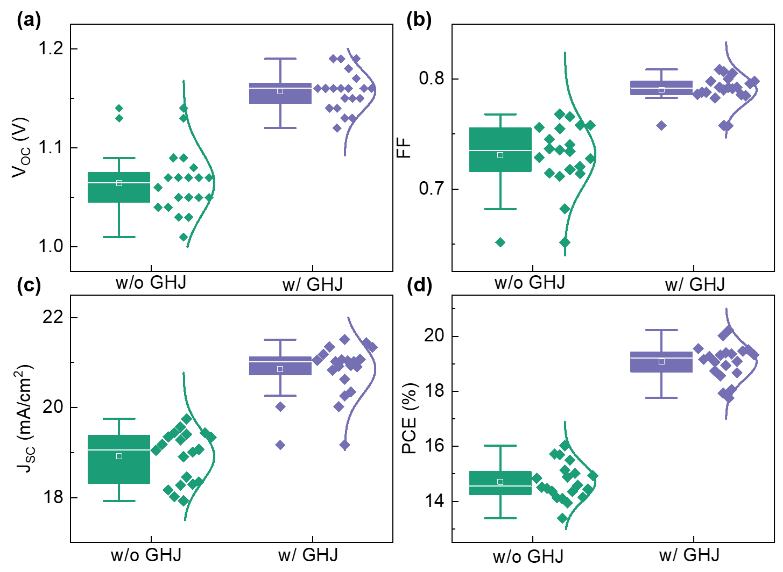


**Fig. S12.** Statistical (a) V_OC_, (b) FF, (c) J_SC_, and (d) PCE of 20 independent WBG PSCs without and with GHJ, respectively.


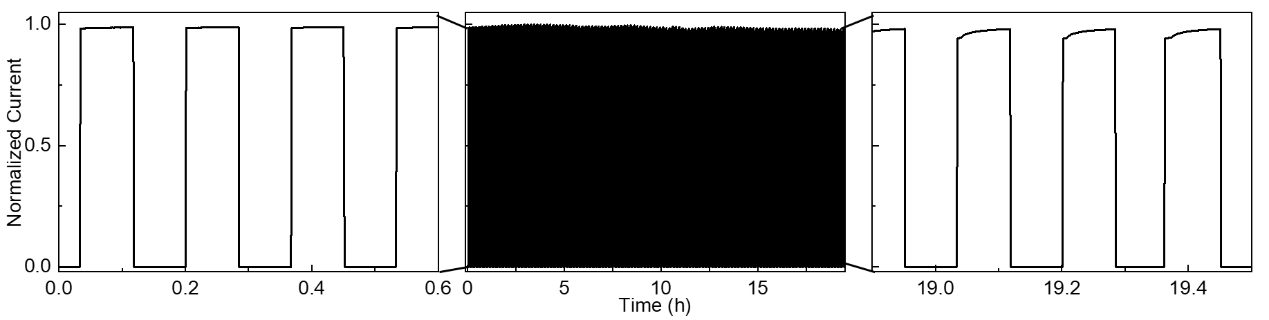


**Fig. S13**. Response properties evaluated with an on/off-modulated 405 nm laser (40 mW cm^−2^) for the typical WBG PSC with GHJ recorded in ambient air.


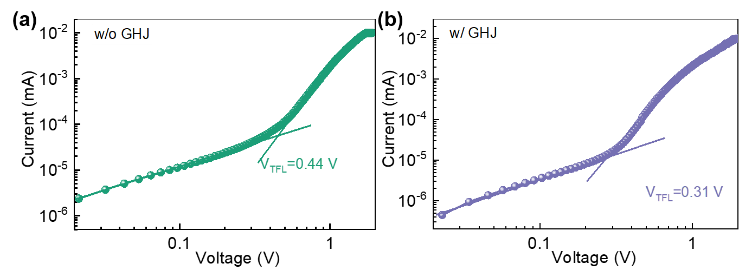


**Fig. S14.** SCLC curves of WBG films (a) without and (b) with GHJ, respectively.


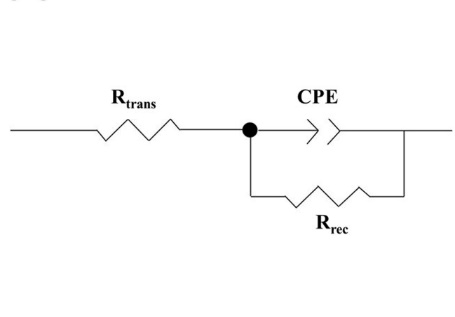


**Fig. S15.** Equivalent circuit model for fitting the Nyquist plots.


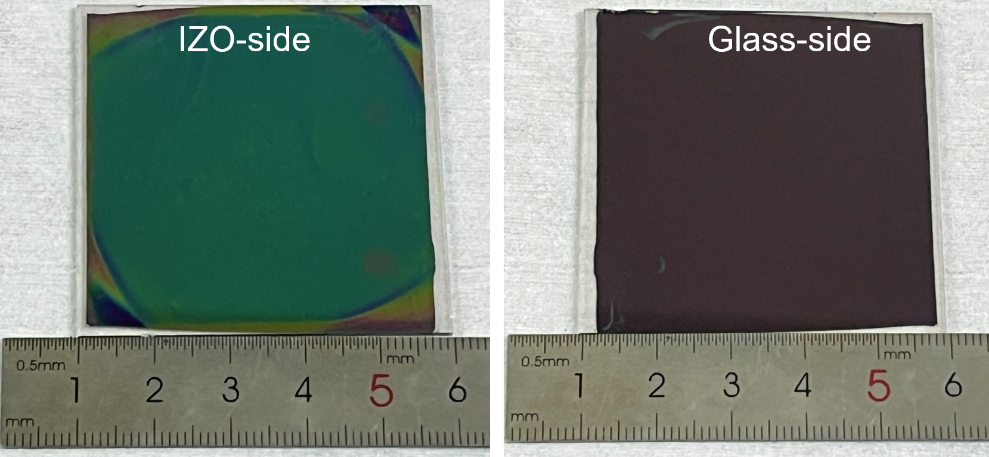


**Fig. S16**. Photograph of large-area semitransparent WBG device used as a filter.


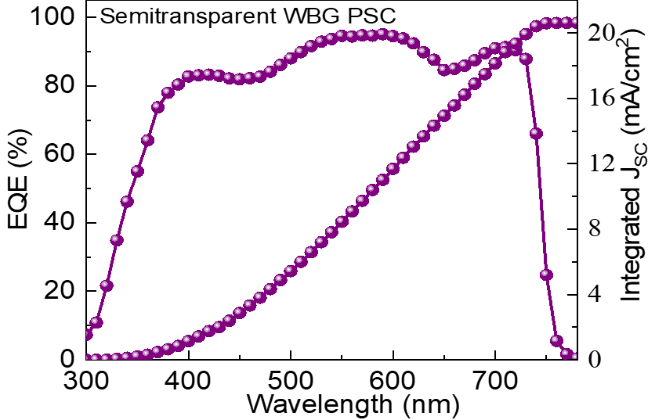


**Fig. S17.** EQE and integrated current of semitransparent WBG PSC.

**Table S1.** Fitting parameters for the TRPL decay of the control sample as well as WBG films with excessive PbI_2_ and GHJ, respectively.

| **Samples** | **A_1_** | **τ_1_** | **A_2_** | **τ_2_** | **τ_ave_** |
| --- | --- | --- | --- | --- | --- |
| control | 2.69 | 12.98 | 0.47 | 135.55 | 92.31 |
| w/ excessive PbI_2_ | 0.50 | 18.61 | 0.51 | 188.81 | 173.81 |
| w/ GHJ | 0.37 | 27.18 | 0.59 | 245.36 | 231.12 |

**Table S2.** Photovoltaic parameters for WBG PSCs without and with GHJ measured by forward and reverse scanning, respectively.

|  |  | **J_SC_ (mA/cm^2^)** | **V_OC_ (V)** | **FF** | **PCE (%)** | **Hysteresis index(%)** |
| --- | --- | --- | --- | --- | --- | --- |
| w/o GHJ | FS | 19.4 | 1.042 | 0.701 | 14.17 | 9.2 |
|  | RS | 19.46 | 1.115 | 0.713 | 15.47 |  |
| w/ GHJ | FS | 21.36 | 1.171 | 0.776 | 19.41 | 3.9 |
|  | RS | 21.39 | 1.181 | 0.799 | 20.18 |  |

**Table S3.** Photovoltaic parameters for WBG PSCs with GHJ in an area of 0.07 and 0.6 cm^2^.

|  | **J_SC_ (mA/cm^2^)** | **V_OC_ (V)** | **FF** | **PCE (%)** |
| --- | --- | --- | --- | --- |
| 0.07 cm^2^ | 21.05 | 1.181 | 0.801 | 19.89 |
| 0.6 cm^2^ | 21.36 | 1.172 | 0.776 | 18.88 |

**Table S4.** Photovoltaic parameters for semitransparent WBG PSC as well as TOPCon silicon solar cells before and after filtering with the semitransparent device.

| **samples** | **J_SC_ (mA/cm^2^)** | **V_OC_ (V)** | **FF** | **PCE (%)** |
| --- | --- | --- | --- | --- |
| TOPCon Si solar cell | 41.61 | 0.72 | 0.751 | 22.50 |
| Filtered TOPCon Si solar cell | 20.63 | 0.70 | 0.747 | 10.78 |
| Semitransparent WBG PSC | 21.40 | 1.16 | 0.811 | 20.13 |
| 4-T tandem solar cell |  |  |  | **30.91** |

**Table S5.** A summary of the state-of-the-art efficiencies of semi-transparent PSCs and 4-T perovskite/silicon tandem devices.

| **Bandgap of top cell (eV)** | | **PCE of Semi-transparent PSC (%)** | **PCE of 4-T tandem device (%)** | **Published year** | **Reference** |
| --- | --- | --- | --- | --- | --- |
| 1.68 | 20.13 | 30.91 |  | **This work** |  |
| 1.65 | 19.9 | 29.8 | 2023 | [1] |  |
| 1.77 | 18.57 | 30.24 | 2022 | [2] |  |
| 1.55 | 20.3 | 29.65 | 2022 | [3] |  |
| 1.65 | 19.28 | 28.28 | 2022 | [4] |  |
| 1.57 | 16.25 | 24.43 | 2022 | [5] |  |
| 1.63 | 18.19 | 27.59 | 2021 | [6] |  |
| 1.55 | 19.53 | 27.7 | 2021 | [7] |  |
| 1.60 | 19.8 | 28.3 | 2021 | [8] |  |
| 1.59 | 18.9 | 26.0 | 2020 | [9] |  |
| 1.63 | 19.0 | 28.2 | 2020 | [10] |  |
| 1.65 | 17.5 | 25.7 | 2020 | [11] |  |
| 1.59 | 18.3 | 27 | 2020 | [12] |  |
| 1.59 | 18.8 | 24.42 | 2020 | [13] |  |
| 1.60 | 17.7 | 24.7 | 2020 | [14] |  |
| 1.70 | 14.8 | 24.2 | 2019 | [15] |  |
| 1.72 | 14.1 | 22.4 | 2019 | [16] |  |
| 1.58 | 17.7 | 25.5 | 2019 | [16] |  |
| 1.59 | 16.7 | 23.1 | 2019 | [17] |  |
| 1.55 | 16.5 | 25.26 | 2018 | [18] |  |
| 1.60 | 16.6 | 22.4 | 2018 | [19] |  |
| 1.60 | 15.3 | 23.9 | 2018 | [20] |  |
| 1.59 | 14.7 | 21.5 | 2018 | [21] |  |
| 1.73 | 16 | 26.4 | 2017 | [22] |  |
| 1.60 | 16.3 | 25.2 | 2016 | [23] |  |
| 1.60 | 17.4 | 24.5 | 2016 | [24] |  |
| 1.59 | 14.2 | 22.6 | 2016 | [25] |  |
| 1.74 | 15.1 | 22.4 | 2016 | [26] |  |
| 1.55 | 12.4 | 20.1 | 2016 | [27] |  |
| 1.60 | 10.36 | 18.18 | 2015 | [28] |  |
| 1.60 | 6.2 | 13.4 | 2015 | [29] |  |
| 1.59 | 12.7 | 17 | 2015 | [30] |  |

**References:**

1. Liu Z, Zhu C, Luo H, Kong W, Luo X, Wu J, Ding C, Chen Y, Wang Y, Wen J, Grain Regrowth and Bifacial Passivation for High‐Efficiency Wide‐Bandgap Perovskite Solar Cells. *Adv. Energy Mater.* 2023; **13**(2), 2203230.

2. Yao Y, Hang P, Li B, Hu Z, Kan C, Xie J, Wang Y, Zhang Y, Yang D, Yu X, Phase‐Stable Wide‐Bandgap Perovskites for Four‐Terminal Perovskite/Silicon Tandem Solar Cells with Over 30% Efficiency. *Small* 2022; **18**(38), 2203319.

3. Abbasiyan A, Noori M, Baghban H, A highly efficient 4-terminal perovskite/silicon tandem solar cells using QIBC and IBC configurations in the top and bottom cells, respectively. *Mater. Today Energy* 2022; **28**, 101055.

4. Chen Y, Ying Z, Li X, Wang X, Wu J, Wu M, Sun J, Sheng J, Zeng Y, Yan B, Self-sacrifice alkali acetate seed layer for efficient four-terminal perovskite/silicon tandem solar cells. *Nano Energy* 2022; **100**, 107529.

5. Jhou J-C, Gaurav A, Chang C-H, Lin C-F, Enhanced efficiency of semitransparent perovskite solar cells via double-sided sandwich evaporation technique for four terminal perovskite-silicon tandem application. *Nano Mater.* 2022; **12**(9), 1569.

6. Ying Z, Yang X, Zheng J, Zhu Y, Xiu J, Chen W, Shou C, Sheng J, Zeng Y, Yan B, Charge-transfer induced multifunctional BCP: Ag complexes for semi-transparent perovskite solar cells with a record fill factor of 80.1%. *J. Mater. Chem. A* 2021; **9**(20), 12009-12018.

7. Kanoun A A, Goumri‐Said S, Kanoun M B, Device design for high‐efficiency monolithic two‐terminal, four‐terminal mechanically stacked, and four‐terminal optically coupled perovskite‐silicon tandem solar cells. *Int. J. Energy Res.* 2021; **45**(7), 10538-10545.

8. Yang D, Zhang X, Hou Y, Wang K, Ye T, Yoon J, Wu C, Sanghadasa M, Liu S F, Priya S, 28.3%-efficiency perovskite/silicon tandem solar cell by optimal transparent electrode for high efficient semitransparent top cell. *Nano Energy* 2021; **84**(105934.

9. Park H H, Kim J, Kim G, Jung H, Kim S, Moon C S, Lee S J, Shin S S, Hao X, Yun J S, Transparent electrodes consisting of a surface‐treated buffer layer based on tungsten oxide for semitransparent perovskite solar cells and four‐terminal tandem applications. *Small Method.* 2020; **4**(5), 2000074.

10. Chen B, Baek S-W, Hou Y, Aydin E, De Bastiani M, Scheffel B, Proppe A, Huang Z, Wei M, Wang Y-K, Enhanced optical path and electron diffusion length enable high-efficiency perovskite tandems. *Nat. Commun.* 2020; **11**(1), 1257.

11. Gharibzadeh S, Hossain I M, Fassl P, Nejand B A, Abzieher T, Schultes M, Ahlswede E, Jackson P, Powalla M, Schäfer S, 2D/3D heterostructure for semitransparent perovskite solar cells with engineered bandgap enables efficiencies exceeding 25% in four‐terminal tandems with silicon and CIGS. *Adv. Funct. Mater.* 2020; **30**(19), 1909919.

12. Wang Z, Zhu X, Zuo S, Chen M, Zhang C, Wang C, Ren X, Yang Z, Liu Z, Xu X, 27%‐Efficiency four‐terminal perovskite/silicon tandem solar cells by sandwiched gold nanomesh. *Adv. Funct. Mater.* 2020; **30**(4), 1908298.

13. Lee C, Lee S-W, Bae S, Shawky A, Devaraj V, Anisimov A, Kauppinen E I, Oh J-W, Kang Y, Kim D, Carbon Nanotube Electrode‐Based Perovskite–Silicon Tandem Solar Cells. *Sol. RRL* 2020; **4**(12), 2000353.

14. Dewi H A, Wang H, Li J, Thway M, Lin F, Aberle A G, Mathews N, Mhaisalkar S, Bruno A, Four‐terminal perovskite on silicon tandem solar cells optimal measurement schemes. *Energy Tech.* 2020; **8**(4), 1901267.

15. Bett A J, Winkler K M, Bivour M, Cojocaru L, Kabakli O z S, Schulze P S, Siefer G, Tutsch L, Hermle M, Glunz S W, Semi-transparent perovskite solar cells with ITO directly sputtered on Spiro-OMeTAD for tandem applications. *ACS Appl. Mater. Interfaces* 2019; **11**(49), 45796-45804.

16. An S, Chen P, Hou F, Wang Q, Pan H, Chen X, Lu X, Zhao Y, Huang Q, Zhang X, Cerium-doped indium oxide transparent electrode for semi-transparent perovskite and perovskite/silicon tandem solar cells. *Sol. Energy* 2020; **196**(409-418.

17. Lee S-W, Bae S, Cho K, Kim S, Hwang J-K, Lee W, Lee S, Hyun J Y, Lee S, Choi S B, Sputtering of TiO_2_ for high-efficiency Perovskite and 23.1% Perovskite/silicon 4-terminal tandem solar cells. *ACS Appl. Energy Mater.* 2019; **2**(9), 6263-6268.

18. Hajjiah A, Parmouneh F, Hadipour A, Jaysankar M, Aernouts T, Light management enhancement for four-terminal perovskite-silicon tandem solar cells: the impact of the optical properties and thickness of the spacer layer between sub-cells. *Materials* 2018; **11**(12), 2570.

19. Ren Z W, Zhou J X, Zhang Y K, Ng A, Shen Q, Cheung S H, Shen H, Li K, Zheng Z J, So S K, Djuric A B, Surya C, Strategies for high performance perovskite/crystalline silicon four-terminal tandem solar cells. *Sol.Energy Mater. Sol. Cell.* 2018; **179**(36-44.

20. Jaysankar M, Filipič M, Zielinski B, Schmager R, Song W, Qiu W, Paetzold U W, Aernouts T, Debucquoy M, Gehlhaar R J E, Science E, Perovskite–silicon tandem solar modules with optimised light harvesting. *Energy Environ. Sci.* 2018; **11**(6), 1489-1498.

21. Kanda H, Shibayama N, Uzum A, Umeyama T, Imahori H, Ibi K, Ito S, Effect of silicon surface for perovskite/silicon tandem solar cells: flat or textured? *ACS Appl. Mater. Interfaces* 2018; **10**(41), 35016-35024.

22. Duong T, Wu Y, Shen H, Peng J, Fu X, Jacobs D, Wang E C, Kho T C, Fong K C, Stocks M, Rubidium multication perovskite with optimized bandgap for perovskite‐silicon tandem with over 26% efficiency. *Adv. Energy Mater.* 2017; **7**(14), 1700228.

23. Werner J, Barraud L, Walter A, Brauninger M, Sahli F, Sacchetto D, Tetreault N, Paviet-Salomon B, Moon S J, Allebe C, Despeisse M, Nicolay S, De Wolf S, Niesen B, Ballif C, Efficient Near-Infrared-Transparent Perovskite Solar Cells Enabling Direct Comparison of 4-Terminal and Monolithic Perovskite/Silicon Tandem Cells. *ACS Energy Lett.* 2016; **1**(2), 474-480.

24. Peng J, Duong T, Zhou X Z, Shen H P, Wu Y L, Mulmudi H K, Wan Y M, Zhong D Y, Li J T, Tsuzuki T, Weber K J, Catchpole K R, White T P, Efficient Indium-Doped TiO_x_ Electron Transport Layers for High-Performance Perovskite Solar Cells and Perovskite-Silicon Tandems. *Adv. Energy Mater.* 2017; **7**(4), 1601768.

25. Jaysankar M, Qiu W, van Eerden M, Aernouts T, Gehlhaar R, Debucquoy M, Paetzold U W, Poortmans J, Four‐terminal perovskite/silicon multijunction solar modules. *Adv. Energy Mater.* 2017; **7**(15), 1602807.

26. McMeekin D P, Sadoughi G, Rehman W, Eperon G E, Saliba M, Hörantner M T, Haghighirad A, Sakai N, Korte L, Rech B, A mixed-cation lead mixed-halide perovskite absorber for tandem solar cells. *Science* 2016; **351**(6269), 151-155.

27. Duong T, Lal N, Grant D, Jacobs D, Zheng P, Rahman S, Shen H, Stocks M, Blakers A, Weber K, Semitransparent perovskite solar cell with sputtered front and rear electrodes for a four-terminal tandem. *IEEE J. Photovolt.* 2016; **6**(3), 679-687.

28. Werner J, Dubuis G, Walter A, Loper P, Moon S J, Nicolay S, Morales-Masis M, De Wolf S, Niesen B, Ballif C, Sputtered rear electrode with broadband transparency for perovskite solar cells. *Sol. Energy Mater. Sol. Cells* 2015; **141**(407-413.

29. Lang F, Gluba M A, Albrecht S, Rappich J r, Korte L, Rech B, Nickel N H, Perovskite solar cells with large-area CVD-graphene for tandem solar cells. *J. Phys. Chem. Lett.* 2015; **6**(14), 2745-2750.

30. Bailie C D, Christoforo M G, Mailoa J P, Bowring A R, Unger E L, Nguyen W H, Burschka J, Pellet N, Lee J Z, Grätzel M, Semi-transparent perovskite solar cells for tandems with silicon and CIGS. *Energy Environ. Sci.* 2015; **8**(3), 956-963.
